# Applying Machine Learning with Localized Surface Plasmon Resonance Sensors to Detect SARS-CoV-2 Particles

**DOI:** 10.3390/bios12030173

**Published:** 2022-03-13

**Authors:** Jiawei Liang, Wei Zhang, Yu Qin, Ying Li, Gang Logan Liu, Wenjun Hu

**Affiliations:** School of Life Science and Technology, Huazhong University of Science and Technology, Wuhan 430074, China; d201980525@hust.edu.cn (J.L.); m201871816@hust.edu.cn (W.Z.); m202172305@hust.edu.cn (Y.Q.); m201971801@hust.edu.cn (Y.L.); loganliu@hust.edu.cn (G.L.L.)

**Keywords:** SARS-CoV-2, machine learning, LSPR sensor, microscopic imaging

## Abstract

The sudden outbreak of COVID-19 rapidly developed into a global pandemic, which caused tens of millions of infections and millions of deaths. Although SARS-CoV-2 is known to cause COVID-19, effective approaches to detect SARS-CoV-2 using a convenient, rapid, accurate, and low-cost method are lacking. To date, most of the diagnostic methods for patients with early infections are limited to the detection of viral nucleic acids via polymerase chain reaction (PCR), or antigens, using an enzyme-linked immunosorbent assay or a chemiluminescence immunoassay. This study developed a novel method that uses localized surface plasmon resonance (LSPR) sensors, optical imaging, and artificial intelligence methods to directly detect the SARS-CoV-2 virus particles without any sample preparation. The virus concentration can be qualitatively and quantitatively detected in the range of 125.28 to 10^6^ vp/mL through a few steps within 12 min with a limit of detection (LOD) of 100 vp/mL. The accuracy of the SARS-CoV-2 positive or negative assessment was found to be greater than 97%, and this was demonstrated by establishing a regression machine learning model for the virus concentration prediction (R^2^ > 0.95).

## 1. Introduction

Achieving a rapid diagnosis of SARS-CoV-2 is important to prevent the spread of the epidemic and enables early intervention in the disease [1]. Most detection techniques, such as polymerase chain reaction (PCR) detection, thermal cycling, isothermal amplification [2,3,4], and serum IgM/IgG antibody detection [5,6,7,8], are time-consuming, expensive and require bulky analytical instrumentation. Moreover, well-trained laboratory professionals and advanced medical infrastructures are required [9]. Therefore, there is an urgent need for point-of-care (POC) detection.

Recently, some studies have applied surface plasmon resonance (SPR) and low-cost local surface plasmon resonance (LSPR) to the rapid detection of SARS-CoV-2 particles [10,11,12,13,14,15]. However, these methods also have limitations in application. For example, virus detection based on SPR usually requires sophisticated and expensive optomechanical systems to monitor the changes in the refraction angle produced by molecular interactions, which limits its large-scale application [15]. Moreover, conventional LSPR devices only extract the global changes in the entire or partial surface optical signal, since most of them only extract simple spectral signals, such as changes in the phase or intensity of spectral peaks, which conceals and ignores substantial rich information regarding the temporal and spatial distribution of the signal changes during detection [10,11,12,13,14]. In short, these limitations will affect their sensitivity, specificity and accuracy in clinical detection. 

A possible solution to the above problems would be to analyze the color image information of LSPR. However, most of the current methods based on LSPR images simply use the change in the gray value of the image, and the extraction of image information is not deep enough [16,17,18]. Therefore, a method that can fully utilize LSPR color image information urgently needs to be established.

In this study, we introduce microscopic color imaging based on nanostructured LSPR sensors, as well as the extraction and deep mining of image features. In addition, machine learning is used to model the features, thereby greatly improving the accuracy, sensitivity, and repeatability, but reducing the detection cost and time during SARS-CoV-2 virus particle detection. This technique is implemented in a one-step direct immunoassay. This assay quantifies the concentration of SARS-CoV-2 in the range of 125.28–10^6^ (virus particles/mL, vp/mL). The virus particles are adsorbed onto the surface of the sensors, and a solution of Au nanoparticles labeled with SARS-CoV-2 mAb is added. When the antibody in the solution recognizes and binds to the spike protein of SARS-CoV-2, it can cause a strong local transmission inhibition in the far field. It results in the reduction of the overall gray value of the color channel in the image where the spectral peak is located, and the degree of reduction is strongly correlated with the concentration of the virus particles. Through further analysis and mining of the image color information, the different hue ratio data of sensor images with a large number of different virus concentrations are input into the machine learning model. Thus, a more accurate, convenient, and inexpensive SARS-CoV-2 detection method is established. Furthermore, we established a support vector machine (SVM) classifier (F1 > 97%) model to determine the presence of a virus and an SVM regression model to predict the virus concentration (R^2^ > 95%). 

## 2. Materials and Methods

### 2.1. Material

The SARS-CoV-2,MERS (Middle East respiratory syndrome) and VSV (vesicular stomatitis virus) pseudovirus samples were obtained from the Shanghai Public Health Clinical Center (Shanghai, China). The SARS-COV-2 antibody-CR3022 (catalog number CHA005) was purchased from Sanyou Biopharmaceutical Co., Ltd. (Shanghai, China). Phosphate-buffered saline (PBS) was purchased from Sigma-Aldrich, and the SARS-COV-2 RBD-mAbs (GHMA 105-1, GHMA 105-2) were purchased from Goodhere Biological Technology Co., Ltd. (Hangzhou, China).

### 2.2. Preparation of the MIM Nanocup Array Device

The Au-titanium dioxide(TiO_2_)-Au layer to form a metal-insulator-metal (MIM) nanocup array device was prepared using a replica molding process. Laser interference lithography was used to fabricate a tapered nanopillar pattern on a quartz substrate. Then, we evenly applied the ultraviolet (UV)-curing polymer (NOA-61, Sigma Aldrich, Shanghai, China) on the mold, with a layer of polyethylene terephthalate (PET) film on top of the mold. After curing with ultraviolet light (105 mW/cm^2^) for 2 min, the periodic nanopore pattern was carefully peeled off from the mold. Titanium (9 nm) and gold (110 nm) were deposited by applying electron-beam evaporation to fabricate the nano-plasma sensors. The resulting nanocup array had a period of 200 nm, a diameter of 200 nm, and a height of 450 nm

### 2.3. Preparation of Au-NPs Labeled with SARS-CoV-2 mAb

First, the pH of the Au-NP colloidal solution was adjusted to 7.2, which was achieved by adding 0.01 M of the dilute K_2_CO_3_ solution. Second, SARS-CoV-2 mAb, prepared in a glycine buffer solution (100 nmol/L) with a pH of 7.0, was added dropwise to 1 mL of the 30 nm colloidal Au-NP solution. Subsequently, the SARS-CoV-2 mAb was incubated with Au-NPs for half an hour. After incubation, 10 μL of 1% BSA diluted in Tris-HCl was added and incubated for 10 min. The Au-NP suspension was centrifuged for 30 min, and the rotation speed was set to 8000 rpm. The centrifugation and resuspension steps were performed three times. In the final resuspension step, the Au-NP pellet was resuspended in 100 μL of 1× Tris-HCl, containing 0.2% *w*/*v* PEG and 0.05% *w*/*v* Tween20. The conjugated AuNPs were stored at 4 °C until further use.

### 2.4. Optical Settings

An Olympus IX73 vertical fluorescence microscope operating in transmission mode was used for the microscopic measurements. A 100 W halogen lamp was used as the light source. The light path included a piece of ground glass, a condenser with an NA of 0.3, and an objective lens with a magnification of 10 times and an NA of 0.3. The RGB color images were captured with a charge-coupled device (CCD) camera that was controlled using the cellSens software. While capturing all the images, all the camera settings remained unchanged. The exposure time was 5 ms, and the RGB gain was set to 1. A spectrometer (iHR320 HORIBA) was used to obtain the transmission spectra. The transmission spectrum from the sample was normalized to the light source spectrum to obtain the final transmission data. The collection of the spectral data was controlled using a customized LabVIEW program. 

### 2.5. Using Au-NP Enhanced Technology to Measure the SARS-CoV-2 Pseudovirus

First, the surface of the sensor was cleaned with 70% ethanol and deionized water. The transmission spectrum and images were obtained in the deionized water, and the collection area was recorded. Next, we added 50 μL of the SARS-CoV-2 pseudovirus solution with different concentrations on the sensor and captured the abovementioned area image again, which was used as the starting point (SP) for the entire experiment. Finally, we added 10 μL of Au-NP-labeled SARS-CoV-2 mAb solution to the sensor and waited for the AuNPs to fully bind to the different epitopes and different antibodies or proteins in the receptor binding (RBD) of the SARS-CoV-2 spike protein. This required approximately 10–12 min, and then we captured the image of the above area again at the end of the reaction.

### 2.6. Software

This study used the following software: Python 3.7, Jupyter notebook compiler, R-3.4.1, R-studio compiler, the RNA-seq processing package limma21,22 based on the R language, the SOM algorithm package Kohonen23 based on the R language, the image processing tool OpenCV for Python, the machine learning tool scikit-learn 0.21.3, the plot package seaborn, the machine learning model evaluation package Yellowbrick, and the rpy2 package that builds the R language into Python

### 2.7. Image R Channel and G Channel Difference Calculation

Using the image function of the PIL library that is based on Python 3.7 to read the acquired image, we split the RGB channels of the image and calculated the grayscale histogram of each channel. After that, we subtracted the gray histogram of the corresponding R, G channel of the 12 min image with the detection antibody added and the SP images with the detection antibody added for 0 min. The maximum value of the above corresponding frequency difference minus the minimum value of the difference was taken as the signal data of each sensor, and the Log10 value of signal data of the different concentrations were further counted and linearly fitted.

### 2.8. Image R Channel and G Channel Ratio Calculation

We directly subtracted the image 12 min and SP images. This was done to obtain the difference image. Cropping was performed on the difference image. The original image size was 1920 × 2448, and it was cropped into 456 images of a size of 100 × 100 images. Thereafter, we split each 100 × 100 image into RGB channels, counted the gray value of each channel separately, and plotted the histogram.

By counting the mean of the corresponding frequency histograms for the R channel and G channel of each small image, each concentration obtained 456 R and G channel means, respectively. Next, we calculated the ratio of the R mean to the G mean for each 100 × 100 image. Then, the ratio of R to G for each concentration was plotted on a bar plot and it was fitted by applying one-variable linear regression.

### 2.9. Calculation Method of Limit of Detection

The calculation of LOD mainly depends on the following steps. First, a fitting curve is established. Fit the value of G/R or Log10(Diff) as dependent variable (*y*) and virus concentration as the independent variable (*x*) to get the fitted curve *f*. Second, calculate the inverse function of the fitted curve, *f*^−1^. Third, determine the average value of *y* (G/R, Log10(Diff)) of the blank samples plus three times the maximum standard deviation obtained among all the experimental points (3σmax). The equation is as follows:

*x*_LOD_ = *f*^−1^(*y*_blank_ + 3σmax) (1)

3σmax: Standard deviation obtained among all the experimental points
*y*_blank_: Average value of negative control signal
*f*^−1^: Inverse of the fitting function
*x*_LOD_: Limit of detection of test substance

### 2.10. Statistics of the H Value for the Sensor Images and Calculation of logFC

First, each set consisted of six images for three sensors (we obtained two images for each sensor after adding the detection Ab for SP and 12 min image). The images were then converted from the RGB format into the HSV format. The converted images were split into three channels and the H channels were retained. The images were again divided into 500 × 500-sized images, and the frequency of the H value of each segmented image was counted and used to create a box chart. 

Second, we standardized the different H frequencies of all the images in the same set and used the standardized H frequencies to create a chart. The column name is the name of each 500 × 500 pixels image, the row name is the different hue degree, and the value is the frequency of the corresponding H degree. Specifically, we input each set of data that was acquired after adding the detection Ab for 0 min and 12min into the limma program based on the R language to standardize the data. Then, we used the standardized data to plot a box chart.

Third, we calculated the ratio of the frequency difference of the H degrees of SP images and 12 min images. Specifically, in each set, we took the standardized value of the different H degree frequencies of all the 500 × 500 pixels SP images as the control group. Thereafter, we took the standardized value of the different H degree frequencies of all the 500 × 500 pixels images that were acquired after the addition of the detection Ab for 12 min as the experimental group. We used the RNA-seq analysis method to count the values for the ratio of the frequency difference of the H degrees after adding the detection Ab for 0 min and 12 min.

### 2.11. Expansion of Data

To increase the number of sets, we applied permutations and combinations to reorganize the data for each concentration. First, in the experiment, 23 sensors were used for each concentration, and 3 sensors were selected randomly to obtain a set of data. As a result, there were a total of C_23^3 combinations, namely 1771 sets. Thus, a total of 8855 sets of data were obtained for five different concentrations.

### 2.12. Feature Selection

The self-organizing feature map (SOM) algorithm was used to explore H with highly similar expression patterns. The SOM cluster was constructed using the Kohonen software package based on R 3.4.2. Specifically, we took the ratio values of 180 H as 180 input features, and input the data into the SOM clustering model to cluster H. By observing the trend of the ratio value of H for each neuron, the ratio value of H can be selected. 

### 2.13. Training and Evaluation of Machine Learning Models

The scikit-learn library and Yellowbrick library were used to train and evaluate the model. Specifically, after obtaining the ratio of the corresponding H value, we transformed it into a two-dimensional data frame, where the row name is the number of each group of data (5 × 1771), and the column name is the corresponding H. Next, we entered the sorted data into the machine learning model. First, we divided the data into training and test sets. We used cross-validation and divided the data into five sets. Four of these were randomly selected for model training. After training, the remaining set was used as a test set.

We applied a logistic regression and SVM classifier to train and evaluate the model by assessing the model’s false positive rate, false negative rate, F1 value, and the receiver operating characteristic (ROC) curve of the test sample. The model was trained using SVM regression and linear regression, and it was evaluated based on R^2^, mean square error (MSE), mean absolute error (MAE), and other indicators of the test sample.

## 3. Results

### 3.1. Fabrication of the Nanoplasmonic Resonance Sensor 

**Figure 1:**
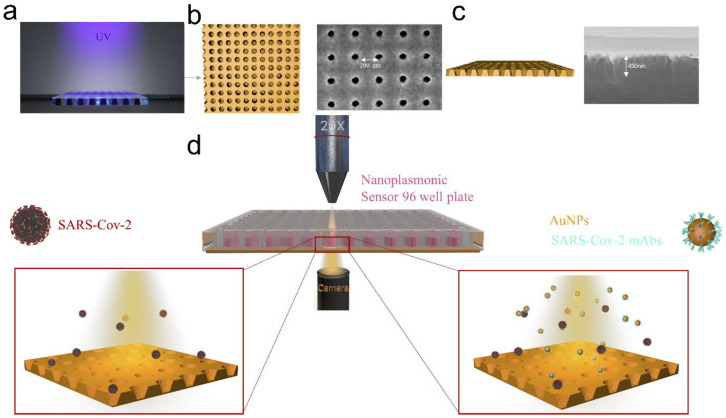
Schematic of SARS-CoV-2 detection with plasmonic sensor chip under the microscope. (**a**) Schematic of the fabrication of Au-TiO_2_-Au nanocup array chip sensor. Spread UV-curable polymer (NOA61) on the PET sheet, solidify the NOA61 on the PET sheet through UV irradiation, deposit metal on the surface of the NOA61 (left). (**b**,**c**) SEM images of the top view of the chip, SEM images of the cross-sectional view of the chip. (**d**) Acquire the microscope image of the chip after adding the SARS-CoV-2 virus (left) and acquire the microscope image of the chip after adding the detection antibody (right).

The sensor was fabricated by combining a metal-insulator-metal multilayer and a 3D nanocup array structure. Figure 1a shows a schematic of the sensor manufacturing process. The UV-curable polymer was evenly spread on the mold with a 220 nm nanocup diameter, 500 nm depth, and 440 nm periodicity. A PET sheet was placed on top of it. After the UV irradiation and polymer curing, the PET was peeled off. Then, a 30 nm titanium dioxide cavity layer and a 90 nm top Au layer were deposited onto it. Figure 1b,c shows a top-down and cross-sectional scanning electron microscope (SEM) image of the device. We can observe that an ordered periodic array was maintained after the entire device was manufactured. Finally, we tested the extinction spectrum of the fabricated chip with water and different concentrations of sucrose solution; we found that the peaks of the extinction spectrum were mainly concentrated in 600 nm (Supplementary Materials Figure S1a).

### 3.2. Image Acquisition of the SARS-CoV-2 Virus Particle Buffer Sensor

A schematic diagram of the SARS-CoV-2 virus particle detection method is shown in Figure 1d. First, a series of SARS-CoV-2 virus particle buffer solutions with a concentration ranging from 0 vp/mL to 10^6^ vp/mL were added (50 μL) onto the sensor. In order to find the change in the image with only virus particles added, we calculated the difference in the frequency of the corresponding gray value of the red channel of the image 12 minutes after the addition and the starting image. It was found that the addition of virus particles caused a frequency change of the gray value corresponding to the red channel of the image, but this change was not meaningful when distinguishing different concentrations, as shown in Figure S1b. Then, the SARS-CoV-2-mAb-labeled Au-NPs were added (10 μL) onto the sensor, and the image was captured again after waiting for 0 min, 5 min and 12 min.

### 3.3. The Changes of the Gray Value of the RGB Channels Can Be Used to Fit the Changes of the SARS-CoV-2 Virus Concentration 

**Figure 2:**
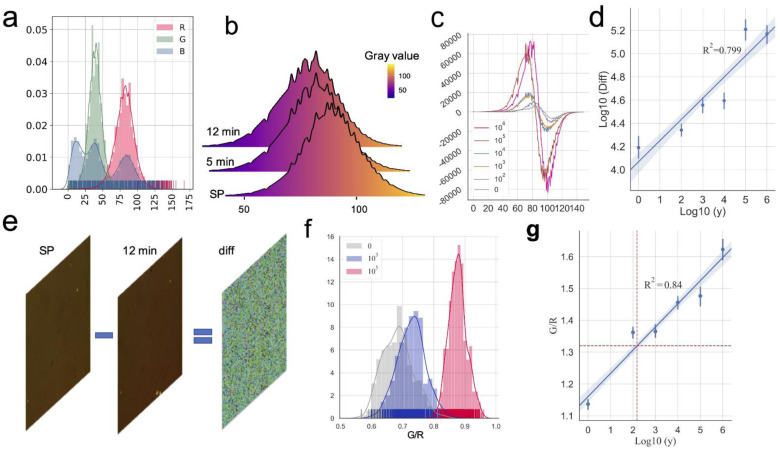
The changes in the gray value of the RGB channels can be used to fit the changes of the SARS-CoV-2 virus concentration. (**a**) The histogram shows the RGB channels gray values count of the chip image after adding the detection antibody. (**b**) The three histograms show the R channel gray values count of chip image at start point (SP), and chip images 5 min and 12 min after adding detection antibody (10^5^ vp/mL). (**c**) The figure shows the difference between the 12 min image and the SP image in each gray frequency of the R channel. (**d**) The fitting result of the linear regression model established by the LogDiff of the different SARS-CoV-2 virus concentrations (R^2^ = 0.799). (**e**) Schematic diagram of obtaining the median of the difference image. Subtract the two images (SP,12 min) to get the difference image. (**f**) The histogram shows the distribution of ratio of G mean to R mean of all cropped difference images at different virus concentrations. (**g**) The fitting result of the linear regression model established by the G/R ratio of all the sets (R^2^ *=* 0.84) The red dotted line of the y-axis is *y*_blank_ + 3σmax, and the red dotted line of the x-axis is LOD.

Initially, we counted the frequency difference between the gray values of the two images, SP and after 12 min (see Section 2). Specifically, we first split the RGB channel of the images for each concentration. When the three channels of the image were split, as shown in Figure 2a, we determined that the gray value of the pixel of the B channel was negligible; thus, the B channel was discarded. Next, we counted the gray value frequency of the SP, 5 min, and 12 min images (10^5^ vp/mL). As shown in Figure 2b, with the prolongation of the reaction time between the spike protein of the virus and the detection antibody, the gray value histogram of the R channel continued to shift to the left. Next, we subtracted the frequency of the corresponding gray value (12 min, SP) in the R and G channels. As shown in Figure 2c and Figure S1c, as the SARS-CoV-2 virus concentration increased, the absolute value of the difference in the frequency of the corresponding gray value increased. Finally, we subtracted the maximum and minimum values of the above difference for the R channel. Then, we took the logarithm to the base 10 and used the obtained value as the signal value of each image to fit the virus concentration. The results are shown in Figure 2d and R^2^ = 0.799, The theoretical limit of detection (LOD) of this method was 347.54 vp/mL. Furthermore, to test the sensitivity and specificity of the device, we also tested MERS and VSV on the device, and we found that the device had a negligible response to the two viruses (Figure S1d). 

The specific manifestation in the difference image was that the ratio of the mean value of the frequency of the R and G gray values changed. Specifically, the SP image and the end point image acquired after adding the detection antibody at 0 min and 12 min were subtracted. As shown in Figure 2e, the difference images were cropped into many 100 × 100 sized images. The gray value frequencies of the R channel and G channel pixels for each 100 × 100 sized image were obtained. Finally, the mean of gray values of the 100 × 100 size image was taken to create a scatter plot, as shown in Figure S1e. As shown in the figure, as the concentration of the SARS-CoV-2 particles increased, the R channel average for the difference image gradually decreased, and the G channel average also decreased. To evaluate this change more accurately, we calculated the ratio of R and G, which was used as a comprehensive index to describe this change. As shown in Figure 2f and Figure S1f, as the concentration of the virus particles increased, the G/R value gradually increased. Finally, we linearly fitted the above ratio data to the virus concentration, as shown in Figure 2g, where R^2^ = 0.843, LOD = 125.28 vp/mL.

### 3.4. Multiple Features Related to SARS-CoV-2 Virus Concentration Can Be Obtained from HSV Format Images 

**Figure 3:**
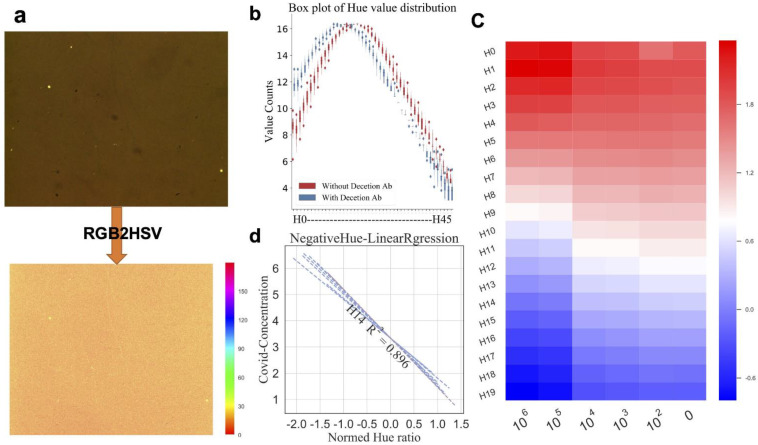
Multiple features related to SARS-CoV-2 virus concentration can be obtained from HSV format images. (**a**) Convert RGB format image of the chip (upper) to HSV format and extract the H channel (bottom). (**b**) The boxplot of frequency of normalized H values of all images of 500 × 500 pixels. Each box represents the distribution of images H frequency. Red box stands for the SP chip, while blue box stands for the 12 min chip (n_500 × 500 = 36) (**c**) Normalized heatmap of ratio values at different degrees of H values in different SARS-CoV-2 concentrations. (**d**) Ordinary linear fitting curves of ratio value at different H degrees with SARS-CoV-2 concentrations; the line represents the fitting curve of a certain H (purple line H14 R^2^ = 0.896).

According to the above results, although we could obtain features that have a certain linear relationship with the change in the SARS-CoV-2 virus concentration, its quasi-merging cannot achieve an ideal effect. For this reason, we further analyzed the image and determined that, by increasing the SARS-CoV-2 virus concentration, this was mainly manifested as color changes in the images. To obtain more color features, we converted the image format into the HSV format, where H stands for the hue, S for the saturation, and V for the brightness. The distribution range of H was 0–360°, and each degree represents a different color; thus, a total of 360 features could be obtained. To save computing resources, we compressed the distribution range from 0–360° to 0–180°, as shown in Figure 3a.

To correct the deviation of the H frequency for the entire image, we cropped the original images of the three sensors in each set to obtain a total of 3 × 12 images of a size of 500 × 500. We then counted the frequency of the H degree for each 500 × 500 sized image and prepared a boxplot (Figure S2a). It was found that there was a certain intra-set difference in the distribution of H in each set. To eliminate this difference, we normalized the data using the limma package of the RNA-seq technology (see Section 2). As shown in Figure S2b, after eliminating the intra-group differences, the H distribution for each small image was consistent. The statistics exhibited a standardized H distribution, as shown in Figure 3b (the x-axis represents different H degrees, and the y-axis represents the corresponding H frequency for the 36 × 500 × 500 sized images). After adding the detection Ab, the distribution of H shifted to the left, and the frequency of H45–160 was negligible. 

Next, we calculated the ratio of the normalized SP image to the 12 min image that corresponded to the mean value of the H frequency. Specifically, we randomly selected a set of data from each concentration and used the RNA-seq analysis method again. We regarded the different degrees of H as a different gene and calculated the average value of the 3 × 12 × 500 × 500 frequency of H at the SP and 12 min that corresponded to a set of data. Finally, we calculated the ratio of the two as a piece of data. As shown in Figure 3c, the ratio increased with the rise in the SARS-CoV-2 virus concentrations for H0–10 and it decreased with an increase in the SARS-CoV-2 concentrations for H10–20. Finally, a number of H ratio values that had a certain linear relationship with the change in the SARS-CoV-2 virus concentration could be obtained, as shown in Figure 3d

### 3.5. Accurate Machine Learning Classifier Models Can Be Obtained from Training Multiple Features

From the above, we obtained the ratios of H0–45 and H160–180 for the different SARS-CoV-2 virus particle concentrations. We used them as image features and inputted them to the machine learning model for training. To avoid the curse of dimensionality, which is caused by too many features, we needed to select the features again. We used the self-organizing neural network method to cluster H and selected the H that increased or decreased as the SARS-CoV-2 virus concentration increased, as shown in Figure S2c.

Machine learning usually requires large data sets. In this study, we found that it was easy to distinguish high-concentration samples from negative samples (Figure 2 and Figure 3), but these data did not contribute to machine learning during identification of low-concentration positive samples. In order to enhance the recognition rate in lower concentrations, we performed a large number of repeated experiments on the SARS-CoV-2 virus particle concentrations of 10^2^, 10^3^, 10^4^, and 10^5^ vp/mL (positive, 1), and 0 vp/mL (negative, 0) to obtain a large amount of data. Before inputting the data into the machine learning model, we adopted a cross-validation method to split the data (see Section 2).

**Figure 4:**
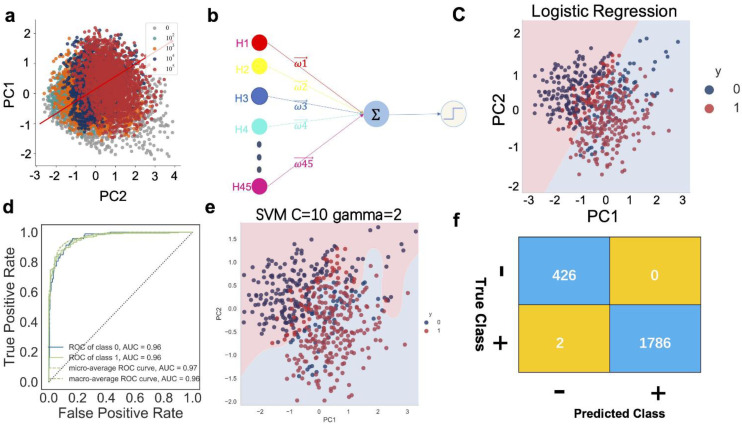
Accurate machine learning classifier models can be obtained from training multiple features. (**a**) Visualization of the variation in different SARS-CoV-2 concentrations by PCA. (**b**) The schematic of inputting the ratio values at different degrees of H in different SARS-CoV-2 concentrations into logistic regression model to predict whether it contains the SARS-CoV-2 virus. (**c**) A schematic diagram of the PC1 and PC2 in logistic regression model. The background color refers to the decision boundary of the classifier (0 refers to the chip image without SARS-CoV-2 virus, and 1 refers to with SARS-CoV-2 virus). (**d**) The ROC and AUC curve of logistic regression, AUC = 0.95, ROC virus positive of class. (**e**) A schematic diagram of the PC1 and PC2 in SVM classifier model. The background color refers to the decision boundary of the classifier (0 refers to the chip image SARS-CoV-2 virus negative, and 1 refers to SARS-CoV-2 virus positive, gamma = 2, C = 10). (**f**) Confusion matrix of SVM classifier (the + refers to virus positive chip image, the − refers to virus negative chip image).

To visualize the spatial distribution of the above data, we applied the principal component analysis (PCA) method to reduce the dimensionality of the data. As shown in Figure 4a, the images of the different SARS-CoV-2 virus particle concentrations had a certain linearity in the space of different H ratios. 

The H ratio data were input into the logistic regression model for training (Figure 4b,c). Finally, a classification model, with a prediction accuracy of 95% or more, was obtained, as demonstrated in Figure 4d. To further improve the accuracy and precision of the model, we adopted the SVM classification model, as shown in Figure 4e,f, and we optimized the parameters by applying the grid search method. Finally, we obtained the optimal value when c = 10 and gamma = 2, as shown in Table S1 and Figure S3. In addition, F1 was 0.9990 and the precision was 0.9986 for the positive model samples.

### 3.6. Accurate Machine Learning Regression Models Can Be Obtained from Training Multiple Features 

**Figure 5:**
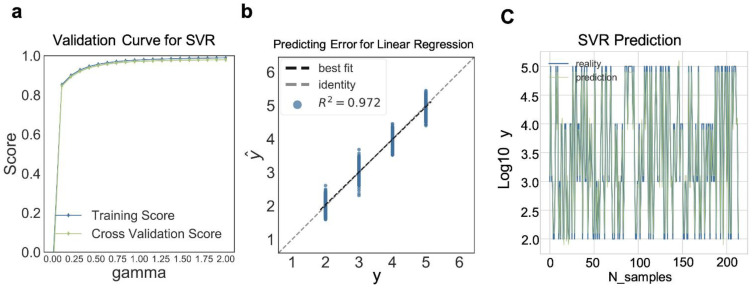
Accurate machine learning regression models can be obtained from training multiple features. (**a**) Validation curve for SVM regressor with increasing gamma (the blue curve stands for training scores, while the green curve stands for cross validation scores). (**b**) Prediction error for SVM regressor (the black line stands for best fit line while the gray line stands for identity line (R^2^ = 0.972)). (**c**) Prediction of SVM regressor (The blue line stands for reality, while the green curve stands for prediction, N_sample = 214).

Finally, we inputted the SARS-CoV-2 positive data (10^2^, 10^3^, 10^4^, 10^5^ vp/mL) into the ordinary linear regression machine learning model for training to predict the concentration of SARS-CoV-2 in the sample, as presented in Table S2. As shown in Figure S3a, as the input features increased, R^2^ also gradually increased (n_features = 56, R^2^ = 0.916). To further improve the accuracy of the prediction and reduce the MSE, we adopted the SVM regression model for training. As shown in Figure 5a,b and Table S2, in comparison to the normal linear regression model mentioned above, the accuracy of the model was greatly improved (R^2^ = 0.972). In terms of the optimization, we continued to use the above optimal parameters. Finally, we determined that the MSE could be stabilized at approximately 100.209, where R^2^ reached 0.983; the prediction for the test data set is shown in Figure 5c. The prediction result for the model was very close to the true value.

## 4. Discussion and Conclusions

In this study, machine learning was applied to LSPR sensor images to detect the SARS-CoV-2 virus. Studies have reported that, as the concentration of the molecules increases, the peak of the absorption spectrum of the LSPR shifts to the right [19,20,21,22]. This suggests that the main change in the microscopic image of the LSPR may be in the color of the image. In our previous studies [23,24,25,26], we determined that, by increasing the concentration of the molecules, the changes in the images were mainly in terms of the R. In this study, we found that the gray value histograms for the R channel of the sensor image shifted to the left (Figure 2b), and when AuNP-Ab was not added, although the frequency of pixels with different gray values would change, it was negligible compared to the AuNP-Ab added (Figure S1b, Figure 2c). We also determined that the G and R channel ratio increased with increase in the SARS-CoV-2 virus concentrations (Figure 2f and Figure S1e), while the signal from the B channel showed no obvious change. This was similar to our previous results, in which the R and G channel pixels of the image changed. Although the above two methods could obtain the image signal value of the fitted virus concentration, the R^2^ of the two was not ideal, and according to the LOD calculation equation [27], the LOD of the two was not ideal (LOD = 347.54, 125.38). This may be because the extracted features were not fine enough. So, more refined image feature extraction methods are in urgent need of development.

Notably, the RNA-seq analysis method played a key role in feature extraction. Generally, the RNA-seq data is standardized during the analysis, mainly to eliminate the noise that is generated during sequencing [28,29]. When we applied this method, we determined that this method played a vital role in eliminating the noise that was generated in the process of plasma microscopic imaging (Figure S2a,b). In addition, we applied this method to analyze the difference in the gene expression in the RNA-seq. This was done to analyze the difference in the H frequency of the HSV format images that were obtained after detection Ab were added, as shown in Figure 3c. The ratio of the H frequency could then be obtained. The ratio of the different H values can be regarded as a set of data to be input into the machine learning model. Figure 4b also shows that the RNA-seq method played a key role in image feature extraction.

There are two core issues that need to be addressed. The first is how to build a labeled LSPR sensor microscopic image dataset that is suitable for machine learning. Data and algorithms are the two core features of artificial intelligence [30,31]. First, we need to determine how to obtain a large number of high-quality image datasets, which are the basis and premise of establishing a machine learning model. In this study, we cropped the images to increase the amount of data. In addition, we arranged and combined the images to increase the amount of data, which was a novel method to expand the dataset. The second problem that needs to be solved is how to avoid overfitting of the model and improve its generalization ability. As previously mentioned, we reduced the number of data dimensions through feature screening and deletion of input data. Specifically, we selected H using SOM [32,33,34] and eliminated some H degrees that had lower frequencies (Figure 3b and Figure S2c). Moreover, when training the model, we reduced overfitting by constraining the complexity of the model. 

To our knowledge, we have applied machine learning to open-field microscopic image analysis while using LSPR sensors for the first time. In addition, we preprocessed and extracted the image features through the RNA-seq method. Then, we inputted the extracted image features, that is, the ratio value of the corresponding H degree, into the machine learning model for training to detect the SARS-CoV-2 virus particles. We used logistic regression to determine whether the LSPR sensor image showed the presence of SARS-CoV-2 particles, as shown in Figure 4b,c. To further improve the prediction accuracy, we used an SVM classification model [35,36], as shown in Figure 4g,f and Table S2. As demonstrated, the method accuracy was much higher than when using only the R, G, and B channels to analyze the image, as illustrated in Figure 2d,g. Finally, we built a regression machine learning model that could predict the concentration of the virus that was contained in the positive samples (Figure 5, Table S2). 

Overall, we have established a new detection method for the SARS-CoV-2 virus. The method is based on the detection of virus particles by LSPR, and imaging and feature extraction of the detection are performed. Next, the extracted features are input into the support vector machine model for training and testing, and finally a model with ideal classification effect is obtained. This method also provides the possibility of low-cost, rapid, and accurate detection of SARS-CoV-2 virus particles in routine clinical environments and resource-limited settings; for example, the image collecting and analyzing systems could both be replaced by cell phones, which is one of our group’s projects. In addition, the method is suitable for many fields, such as the detection of disease biomarkers, as well as drug detection.

## Data Availability

The image dataset used in this study can be found in ADrive (https://www.adrive.com/public/vntUhR, accessed on 9 March 2022), password: 123456.

## Supplementary Materials

The following supporting information can be downloaded at: https://www.mdpi.com/article/10.3390/bios12030173/s1, Figure S1: Linear changes of image features in RGB format, Figure S2: The acquisition and standardization of multi-features of image in HSV format, Figure S3: The training progress and result of SVM classifier, Table S1: Evaluation parameters of SVM Classifier models, Table S2: Evaluation parameters of Regression models.

## References

[1] Poon L.L.M., Peiris M. (2020). Emergence of a Novel Human Coronavirus Threatening Human Health. Nat. Med..

[2] Corman V.M., Landt O., Kaiser M., Molenkamp R., Meijer A., Chu D.K., Bleicker T., Brünink S., Schneider J., Schmidt M.L. (2020). Detection of 2019 Novel Coronavirus (2019-NCoV) by Real-Time RT-PCR. Eurosurveillance.

[3] Tahamtan A., Ardebili A. (2020). Real-Time RT-PCR in COVID-19 Detection: Issues Affecting the Results. Expert. Rev. Mol. Diagn..

[4] Yan C., Cui J., Huang L., Du B., Chen L., Xue G., Li S., Zhang W., Zhao L., Sun Y. (2020). Rapid and Visual Detection of 2019 Novel Coronavirus (SARS-CoV-2) by a Reverse Transcription Loop-Mediated Isothermal Amplification Assay. Clin. Microbiol. Infect..

[5] Corman V.M., Müller M.A., Costabel U., Timm J., Binger T., Meyer B., Kreher P., Lattwein E., Eschbach-Bludau M., Nitsche A. (2012). Assays for Laboratory Confirmation of Novel Human Coronavirus (HCoV-EMC) Infections. Eurosurveillance.

[6] Datta P.K., Liu F., Fischer T., Rappaport J., Qin X. (2020). SARS-CoV-2 Pandemic and Research Gaps: Understanding SARS-CoV-2 Interaction with the ACE2 Receptor and Implications for Therapy. Theranostics.

[7] Fabiani L., Saroglia M., Galatà G., De Santis R., Fillo S., Luca V., Faggioni G., D’Amore N., Regalbuto E., Salvatori P. (2021). Magnetic Beads Combined with Carbon Black-Based Screen-Printed Electrodes for COVID-19: A Reliable and Miniaturized Electrochemical Immunosensor for SARS-CoV-2 Detection in Saliva. Biosens. Bioelectron..

[8] Loeffelholz M.J., Tang Y.-W. (2020). Laboratory Diagnosis of Emerging Human Coronavirus Infections—The State of the Art. Emerg. Microbes. Infect..

[9] Campbell K., Haughey S.A., van den Top H., van Egmond H., Vilariño N., Botana L.M., Elliott C.T. (2010). Single Laboratory Validation of a Surface Plasmon Resonance Biosensor Screening Method for Paralytic Shellfish Poisoning Toxins. Anal. Chem..

[10] Huang L., Ding L., Zhou J., Chen S., Chen F., Zhao C., Xu J., Hu W., Ji J., Xu H. (2021). One-Step Rapid Quantification of SARS-CoV-2 Virus Particles via Low-Cost Nanoplasmonic Sensors in Generic Microplate Reader and Point-of-Care Device. Biosens. Bioelectron..

[11] Behrouzi K., Lin L. (2022). Gold Nanoparticle Based Plasmonic Sensing for the Detection of SARS-CoV-2 Nucleocapsid Proteins. Biosens. Bioelectron..

[12] Masterson A.N., Muhoberac B.B., Gopinadhan A., Wilde D.J., Deiss F.T., John C.C., Sardar R. (2021). Multiplexed and High-Throughput Label-Free Detection of RNA/Spike Protein/IgG/IgM Biomarkers of SARS-CoV-2 Infection Utilizing Nanoplasmonic Biosensors. Anal. Chem..

[13] Qiu G., Gai Z., Saleh L., Tang J., Gui T., Kullak-Ublick G.A., Wang J. (2021). Thermoplasmonic-Assisted Cyclic Cleavage Amplification for Self-Validating Plasmonic Detection of SARS-CoV-2. ACS Nano.

[14] Qiu G., Gai Z., Tao Y., Schmitt J., Kullak-Ublick G.A., Wang J. (2020). Dual-Functional Plasmonic Photothermal Biosensors for Highly Accurate Severe Acute Respiratory Syndrome Coronavirus 2 Detection. ACS Nano.

[15] Yano T.-A., Kajisa T., Ono M., Miyasaka Y., Hasegawa Y., Saito A., Otsuka K., Sakane A., Sasaki T., Yasutomo K. (2022). Ultrasensitive Detection of SARS-CoV-2 Nucleocapsid Protein Using Large Gold Nanoparticle-Enhanced Surface Plasmon Resonance. Sci. Rep..

[16] Cetin A.E., Coskun A.F., Galarreta B.C., Huang M., Herman D., Ozcan A., Altug H. (2014). Handheld High-Throughput Plasmonic Biosensor Using Computational on-Chip Imaging. Light Sci. Appl..

[17] Puiu M., Bala C. (2016). SPR and SPR Imaging: Recent Trends in Developing Nanodevices for Detection and Real-Time Monitoring of Biomolecular Events. Sensors.

[18] Coskun A.F., Cetin A.E., Galarreta B.C., Alvarez D.A., Altug H., Ozcan A. (2014). Lensfree Optofluidic Plasmonic Sensor for Real-Time and Label-Free Monitoring of Molecular Binding Events over a Wide Field-of-View. Sci. Rep..

[19] Ahn H., Song H., Choi J.-R., Kim K. (2017). A Localized Surface Plasmon Resonance Sensor Using Double-Metal-Complex Nanostructures and a Review of Recent Approaches. Sensors.

[20] Meyer M.H.F., Hartmann M., Keusgen M. (2006). SPR-Based Immunosensor for the CRP Detection--a New Method to Detect a Well Known Protein. Biosens. Bioelectron..

[21] Liu Y.-B., Zhai T.-T., Liang Y.-Y., Wang Y.-B., Xia X.-H. (2019). Gold Core-Satellite Nanostructure Linked by Oligonucleotides for Detection of Glutathione with LSPR Scattering Spectrum. Talanta.

[22] Tian Y., Chen Y., Chen M., Song Z.-L., Xiong B., Zhang X.-B. (2021). Peroxidase-like Au@Pt Nanozyme as an Integrated Nanosensor for Ag+ Detection by LSPR Spectroscopy. Talanta.

[23] Hu W., Dang T., Li Z., Lei L., Wang G., Li Y., Xu H., Zhou Z., Liu G.L. (2019). C-Reaction Protein Detection in Human Saliva by Nanoplasmonic Color Imaging. J. Biomed. Nanotechnol..

[24] Gartia M.R., Hsiao A., Sivaguru M., Chen Y., Liu G.L. (2011). Enhanced 3D Fluorescence Live Cell Imaging on Nanoplasmonic Substrate. Nanotechnology.

[25] Wang X., Chang T.-W., Lin G., Gartia M.R., Liu G.L. (2017). Self-Referenced Smartphone-Based Nanoplasmonic Imaging Platform for Colorimetric Biochemical Sensing. Anal. Chem..

[26] Zhang W., Dang T., Li Y., Liang J., Xu H., Liu G.L., Hu W. (2021). Digital Plasmonic Immunosorbent Assay for Dynamic Imaging Detection of Protein Binding. Sens. Actuators B Chem..

[27] Chiavaioli F., Gouveia C.A.J., Jorge P.A.S., Baldini F. (2017). Towards a Uniform Metrological Assessment of Grating-Based Optical Fiber Sensors: From Refractometers to Biosensors. Biosensors.

[28] Diboun I., Wernisch L., Orengo C.A., Koltzenburg M. (2006). Microarray Analysis after RNA Amplification Can Detect Pronounced Differences in Gene Expression Using Limma. BMC Genom..

[29] Ritchie M.E., Phipson B., Wu D., Hu Y., Law C.W., Shi W., Smyth G.K. (2015). Limma Powers Differential Expression Analyses for RNA-Sequencing and Microarray Studies. Nucleic Acids Res..

[30] Deo R.C. (2015). Machine Learning in Medicine. Circulation.

[31] MacEachern S.J., Forkert N.D. (2021). Machine Learning for Precision Medicine. Genome.

[32] Flanagan J.A. (1996). Self-Organisation in Kohonen’s SOM. Neural Netw..

[33] Furukawa T. (2009). SOM of SOMs. Neural Netw..

[34] Blum A.L., Langley P. (1997). Selection of Relevant Features and Examples in Machine Learning. Artif. Intell..

[35] Cherkassky V., Ma Y. (2004). Practical Selection of SVM Parameters and Noise Estimation for SVM Regression. Neural Netw..

[36] Schuldt C., Laptev I., Caputo B. Recognizing Human Actions: A Local SVM Approach. Proceedings of the 17th International Conference on Pattern Recognition.

