# Supplementary material for: Applying Machine Learning with Localized Surface Plasmon Resonance Sensors to Detect SARS-CoV-2 Particles
Source: Biosensors (Basel). 2022 Mar 13;12(3):173. doi: 10.3390/bios12030173 (PMC8946137; doi:10.3390/bios12030173)
Supplement: Supplementary file 1 [file biosensors-12-00173-s001.zip › biosensors-1606334-SI.pdf]

Supporting Information

Figure.S1

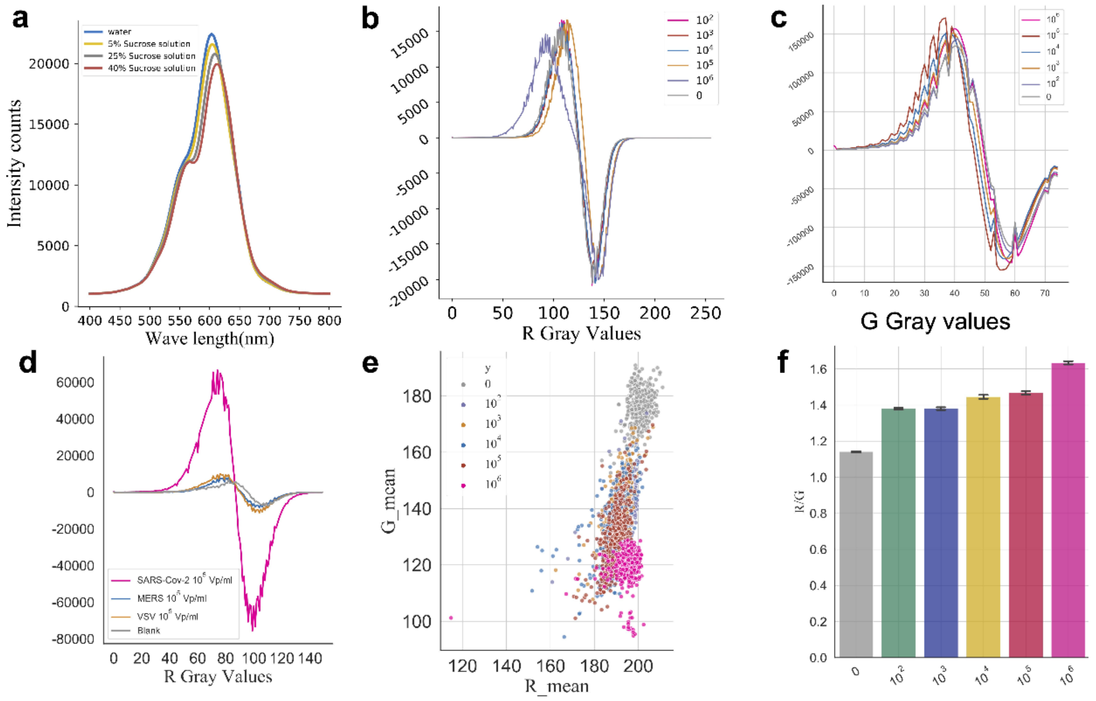

Figure S1. Linear changes of image features in RGB format. (**a**) extinction spectrum of the fabricated chip with water and different concentrations of sucrose solution. (**b**)The figure shows the difference between the 12 min image and the SP image in each gray frequency of the R channel in the chip without AuNPs-Ab added . (**c**)The figure shows the difference between the 12 min image and the SP image in each gray frequency of the G channel. (**d**) The figure shows the difference between the 12 min image and the SP image in each gray frequency of the R channel of different virus. (**e**) The scatterplot shows the distribution of G mean to R mean of all cropped difference images at different SARS-CoV-2 virus concentrations. (**f**) The histogram shows the ratio of G mean to R mean of all cropped difference images at different SARS-CoV-2 virus concentrations.

Figure.S2

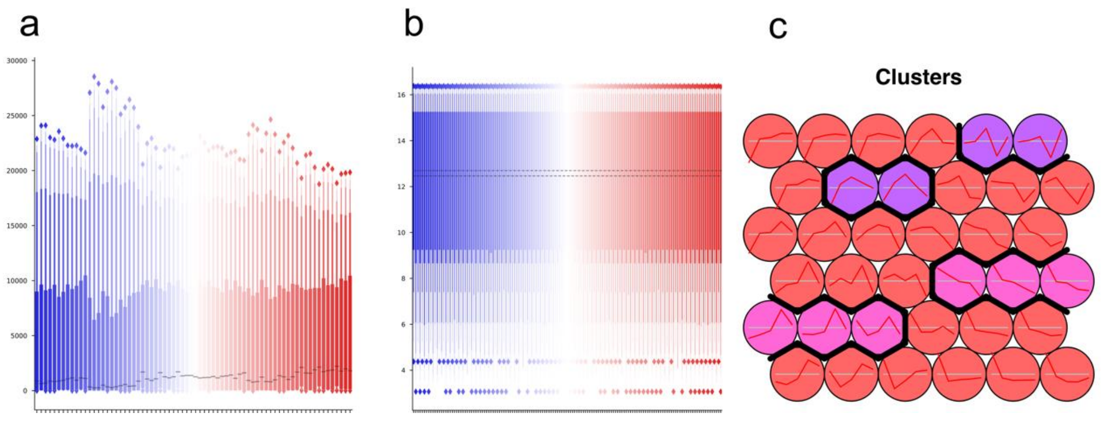

Figure S2. The acquisition and standardization of multi-features of image in HSV format. (a-b) Normalize data by the limma packages of RNA-seq technology, **a** refers to data without standardization, **b**, refers to data with standardization.（Each box stands for the H value distribution of an 500 × 500 pixel image (n_box=72, blue box refers to SP images, red box refers to images at 12 min with SARS-CoV-2 concentration 10^5 vp/ml)）(c) The expression trend of each neuron H ratio in the samples (Neuron 1-36 , from bottom to top, from left to right).

Figure.S3

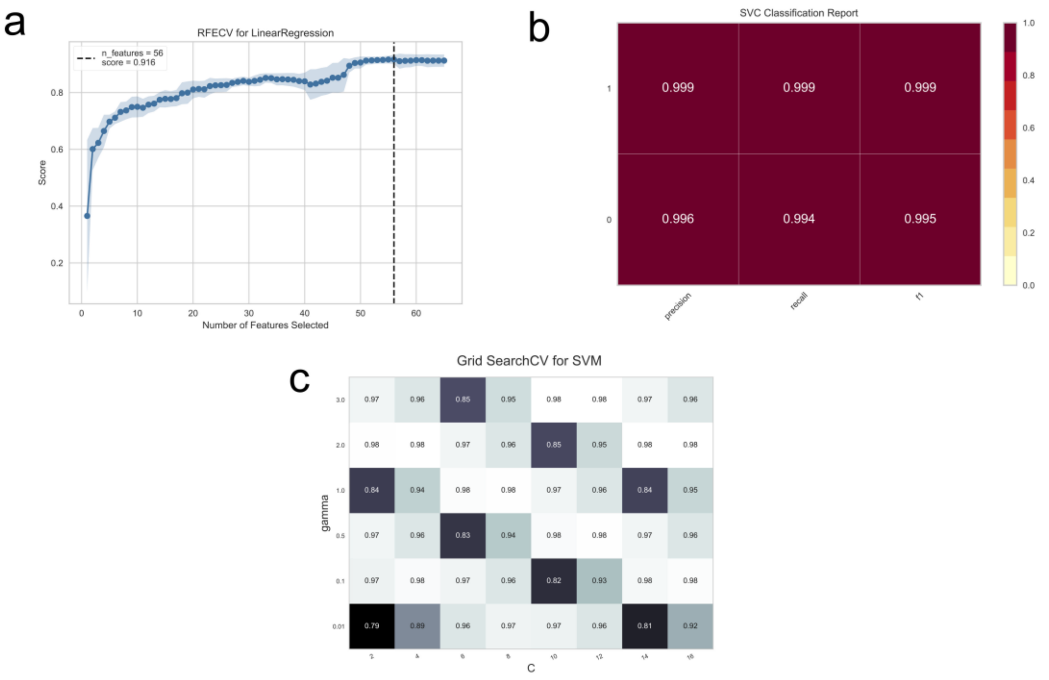

Figure S3. The training progress and result of SVM classifier. (a) The line shows the result of RFECV for linear regression. (b) The heatmap shows the result of SVM classifier. (c) The heatmap of searching the best parameters of SVM classifier by Grid search and Cross-validation (C=16, gamma=2, cv=5)

Table S1. Evaluation parameters of SVM Classifier models

parameters	SVM Classifier Base on different Hue degree Ratio	
	positive	Negative
Precision(95% CI)	0.9986(0.997 , 1)	0.9968(0.991 , 1)
Recall (95% CI)	0.9984(0.997 , 1)	0.9946(0.989 , 1)
F1 (95% CI)	0.9990(0.998 , 1)	0.9948(0.989 , 1)

Table S2. Evaluation parameters of Regression models

parameters	Base on different Hue degree Ratio	
	Linear Regression	SVM
MSE(95% CI)	0.0759(0.0713 , 0.0801)	0.0209(0.0193 , 0.0220)
MAE(95% CI)	0.0220(0.2138 , 0.2254)	0.1074(0.1089 , 0.1054)
R^2 (95% CI)	0.9392(0.9353 , 0.9436)	0.9832(0.9819 , 0.9845)
